# Monitoring child survival in ‘real time’ using routine health facility records: results from Malawi

**DOI:** 10.1111/tmi.12167

**Published:** 2013-08-01

**Authors:** Agbessi Amouzou, Willie Kachaka, Benjamin Banda, Martina Chimzimu, Kenneth Hill, Jennifer Bryce

**Affiliations:** 1 Institute for International Programs, Johns Hopkins Bloomberg School of Public HealthBaltimore, MD, USA; 2 Malawi National Statistical OfficeZomba, Malawi

**Keywords:** child mortality, Health Management Information Systems, Millennium Development Goal, child mortality monitoring

## Abstract

**Objectives:**

Few developing countries have the accurate civil registration systems needed to track progress in child survival. However, the health information systems in most of these countries do record facility births and deaths, at least in principle. We used data from two districts of Malawi to test a method for monitoring child mortality based on adjusting health facility records for incomplete coverage.

**Methods:**

Trained researchers collected reports of monthly births and deaths among children younger than 5 years from all health facilities in Balaka and Salima districts of Malawi in 2010–2011. We estimated the proportion of births and deaths occurring in health facilities, respectively, from the 2010 Demographic and Health Survey and a household mortality survey conducted between October 2011 and February 2012. We used these proportions to adjust the health facility data to estimate the actual numbers of births and deaths. The survey also provided ‘gold-standard’ measures of under-five mortality.

**Results:**

Annual under-five mortality rates generated by adjusting health facility data were between 35% and 65% of those estimated by the gold-standard survey in Balaka, and 46% and 50% in Salima for four overlapping 12-month periods in 2010–2011. The ratios of adjusted health facility rates to gold-standard rates increased sharply over the four periods in Balaka, but remained relatively stable in Salima.

**Conclusions:**

Even in Malawi, where high proportions of births and deaths occur in health facilities compared with other countries in sub-Saharan Africa, routine Health Management Information Systems data on births and deaths cannot be used at present to estimate annual trends in under-five mortality.

## Introduction

Accurate and timely estimates of under-five mortality are essential for evaluating the impact of child survival interventions and for monitoring national and global progress towards the fourth Millennium Development Goal (Millennium Development Goals Indicators 2012). In most low- and middle-income countries (LMICs), vital registration systems are defective or non-existent and cannot provide the data needed (Mahapatra *et al*. 2007; Setel *et al*. 2007). National Health Management Information Systems (HMIS), which are supposed to provide timely health data to support programme monitoring and decision-making, only record events in health facilities, are generally of poor quality and do not include sufficient data to estimate childhood mortality (AbouZahr … Boerma 2005; Commission on Information … Accountability for Women's … Children's Health 2011). Most LMICs therefore rely on household surveys such as the Demographic and Health Surveys (DHS) (Demographic … Health Surveys 2012) and Multiple Indicator Cluster Surveys (MICS) (Multiple Indicator Cluster Surveys 2012) that include a full birth history of women aged 15–49 to estimate levels and trends of under-five mortality. However, these surveys produce estimates that are usually averages over the 5 years before the survey for national-level estimates, and 10 years before the survey for subnational-level estimates, limiting their value for programme monitoring and evaluation.

The Institute for International Programs at Johns Hopkins University is working with African institutions to implement the ‘real-time’ mortality monitoring (RMM) project in five African countries: Ethiopia, Ghana, Malawi, Mali and Niger. The objective of the project is to develop and test locally appropriate and affordable methods for tracking child mortality that can provide valid estimates for recent 12-month periods. In each country, an initial consultative process involving the Ministry of Health and other partners led to the identification of at least two potential methods to be tested. Working with in-country institutional partners, we implement each method for at least 12 months and compare the child mortality rates reported to those obtained by a high-quality (‘gold standard’) census or household survey that includes a full birth history of women aged 15–49. The criterion for judging a method to be successful is that the under-five mortality estimates it produces should not differ from the ‘gold-standard’ estimates by more than 20%.

Although data provided by HMIS are not generally reliable in many countries, the system represents a unique platform for testing a mortality-monitoring approach by assessing whether the data provided can be adjusted to produce valid estimates of child mortality. In addition to problems with data completeness, accuracy and reliability, HMIS data are also subject to selection biases reflecting differential access to health facilities across socio-economic and cultural groups, as well as variable distances between households and health facilities. Even in an ideal situation where data completeness, accuracy and reliability are drastically improved, selection bias will remain an issue, especially in countries where health facility use is low. Most HMISs record data on births and deaths that occur in health facilities. One potential RMM method is to use data on births and under-five deaths recorded by health facilities, and adjust them for any omission or selection bias by calibrating the data to the total population using the proportions of such births and deaths reported in a household survey to have occurred in health facilities. The total number of under-five deaths in a year in a population equals the number of such deaths recorded as occurring in health facilities divided by the proportion of all such deaths that are reported to have occurred in health facilities. The same logic applies for births. Thus, knowledge of the proportion of all births and deaths in a year recorded in health facilities and of the total number of births and deaths thus recorded would allow accurate and timely estimation of annual child mortality rates in the population. If the HMIS accurately records all births and deaths in facilities, the recorded number of births and deaths will be equal to the number of births and deaths that occurred in facilities. The needed proportions of births and under-five deaths occurring in facilities can in principle be obtained from a household survey with a full birth history that records where each birth and under-five death took place.

In a population where the proportions of events occurring in facilities change little over time, these adjustments can be applied to health facility data for real-time mortality estimation. Murray *et al*. have proposed that hospital records can be used to estimate cause-specific mortality fractions at population level, but only in settings where International Classification of Diseases (ICD) codes are used both for records in facilities and in an available vital registration system (Murray *et al*. 2007). Using facility records to estimate all-cause under-five mortality is likely to be applicable in a greater number of countries, but does require that substantial proportions of births and of child deaths occur in health facilities.

Few studies have attempted to use HMIS data because of quality limitations, but there have been attempts to adjust the data provided by the system to obtain accurate national- or subnational level indicators or to link health facility- and population-level data. Using geostatistical modelling, HMIS data organised in space and time have been interpolated to take into account missing data records and used to estimate levels of health facility utilisation and trends in the proportion of health facility visits that are due to specific diseases such as malaria (Gething *et al*. 2006, 2007a,b, 2008). These data ‘kriging’ techniques have been shown to be very reliable and to have only minimal bias (Gething *et al*. 2007a). These methods, however appealing, still have two main drawbacks. First, they are good for imputing missing data, but do not solve the omission and selection bias issues in the data set. Interpretation of findings must therefore be conducted with the caveat that these findings cannot be generalised to the entire population. Second, these techniques are likely to be too advanced for local HMIS officers to apply without strong technical support. Another study conducted at three INDEPTH-Network's demographic surveillance sites assessed the feasibility of recording linkages between health facilities and the population under surveillance using biometric fingerprints (Serwaa-Bonsu *et al*. 2010). The authors concluded that fingerprinting was entirely feasible, although enrolment for fingerprinting was much lower for children than for adults. Although appealing, this approach would not be appropriate for child mortality estimation due to low enrolment rates for children.

We selected Malawi as the most promising setting in which to test this RMM method across the five project countries based on the proportion of births occurring in health facilities in the most recent DHS survey at the time we designed the project in 2009. Best available estimates at that time were that nearly 70% of births in Malawi (69.1%) occurred in health facilities (DHS 2004), compared with 5.8% in Ethiopia (DHS 2005), 58.0% in Ghana (DHS 2008), 47.3% in Mali (DHS 2006) and 18.6% in Niger (DHS 2006) (STATcompiler 2012). The Ministry of Health in Malawi also requested a test of this method because they are working to strengthen their HMIS and facility reporting of vital events, and because they believed it would provide information useful for a new cadre of district-level HMIS officers deployed in 2009.

## Methods

### Setting

We selected two of the 28 districts in Malawi for the test of RMM approaches based on the criteria of high under-five mortality, high fertility, easy access for the study team, full coverage of community health workers deployed and average population size based on the distribution of district population size across the country (Appendix S1). Table 1 shows selected demographic and health system characteristics of the two districts – Balaka in the southern region and Salima in the central region. According to the 2008 Malawi Population Census, Balaka had a population of 316 748 and Salima 340 327. Both districts have high mortality among children under 5 years of age and high fertility (Malawi National Statistical Office (NSO) 2008; National Statistical Office (NSO) … ICF Macro 2011).

**Table 1:** Selected demographic and health system characteristics of Balaka and Salima districts, Malawi

Characteristic (source)	Balaka district	Salima district
Demographic		
Region	South	Central
Population (Census 2008)	316 748	340 327
Under-five mortality rate (DHS 2010)	125	150
Total fertility rate (DHS 2010)	6.0	6.6
Health system		
Number of hospitals (MOH)	1	1
Number of health centres (MOH)		
Public	8	14
Private*	13	8
Number of health surveillance assistants (community health workers)	273	334

* Include health centres run by the Christian Health Association (CHAM) at subsidised rates (6 facilities in Balaka and 5 in Salima).

### Project implementation, data collection and analysis

Before rollout, the RMM project was presented and discussed with stakeholders at national level and in the selected districts. The national-level stakeholders included MOH representatives and other partners involved in maternal, newborn and child health programmes in the country. At district level, the district health Office, the district assembly and some traditional authorities participated in orientation and discussion sessions. A small advisory group was established to provide guidance and ensure that study procedures were consistent with standard operating procedures and not duplicative or burdensome to district staff.

In preparation for the study, the research team and district HMIS officers reviewed the HMIS database of births and deaths and visited all public and private health facilities in each of the two RMM districts to inspect available records of births and deaths.

Current HMIS procedures call for recording of all births and deaths that occur in health facilities, including private facilities. Tallies of deliveries and births are collected every quarter from all health facilities with a maternity ward by the HMIS officers and compiled at district level before being sent to the national level. Cause of death information is recorded only for inpatient deaths. Deaths are not recorded systematically in health centres with no inpatient wards. HMIS forms (Appendix S2) do not allow breakdown of deaths by age.

We developed a short form (Appendix S3) and trained the two district HMIS officers and facility staff to record deaths by age, disaggregated by neonatal, infant and child deaths. There was one district-level HMIS officer in each district, who works with the health centre data clerks or incharges. They were given one-day training on how to fill out the form and transmit the data to the National Statistical Office. They were then provided with monthly incentives of about US$30 as motivation for the extra requirement of disaggregating the deaths by age.

Given that our interest was in assessing the level of reporting of births and deaths within the HMIS system, we did not attempt to modify the existing HMIS recording system for births and deaths.

The HMIS officers visited each health facility every month to extract these data from the health facility records and transfer them to the research team at the National Statistical Office. Data collection began in January 2010 and continued through December 2011.

The basis of this RMM method is the tautology that the true number of events (births or under-five deaths) in a period is equal to the number of events recorded divided by the proportion of all events that were reported. The number of events recorded is known, but the proportion is not. In the case of births, we estimate this proportion as the proportion of births in the past 2 years preceding the survey reported as occurring in a health facility for each district in the 2010 Demographic and Health Survey. However, the 2010 DHS did not record place of death. To apply the method, a question on place of death was included in the full birth history module of a mortality survey conducted in the two districts in late 2011 and early 2012. The objectives of this survey were twofold: to provide the needed proportion of deaths occurring in facilities and to provide ‘gold-standard’ estimates of child mortality against which to assess the performance of this and other RMM methods tested in the two districts. The ‘gold-standard’ survey sampled 12 000 households in each of the two RMM districts. Data were collected between 24 October 2011 and 17 February 2012. We used the 2008 population census frame to select the primary sampling units or enumeration areas (EA) for the survey, with probability proportional to size. Households were selected at a second sampling stage after a complete update of the list of households in each selected EA was conducted. We stratified the sample by district and applied sampling weights during analysis to ensure the representativeness of the results. Interviews were conducted with all women aged 15–49 to obtain a full birth history, that is, the date of birth, survival status, and for children who had died, age at death for each live birth the woman had ever had in her lifetime. We used these data to develop estimates of under-five mortality by dividing under-five deaths by births for four overlapping 12-month periods beginning in January, April, July and October 2010. We computed corresponding sampling errors using the jackknife resampling method and derived 95% confidence intervals (Lohr 1999). Interviewers also asked each woman who reported a child death where the death occurred, with response options of ‘home’, ‘health facility’ or ‘other’. The category ‘other’ included events that occur outside the home and a health facility, for example when a child died outside the home while being sent to a health facility. Two clerks entered the data independently; discrepancies were reconciled through reference to the original survey forms. We used CSPro 4.1 for data entry and STATA 12.1 for further cleaning and analysis. Full details of the survey methods and quality control mechanisms are included in Appendix S4.

We applied the average proportions of births and deaths reported in the surveys to have occurred in health facilities in the years 2009 and 2010 for births and in the years 2010 and 2011 for deaths to the health facility data on births and deaths to estimate the annual number of births and under-five deaths in each district. We used the adjusted numbers of events to compute under-five mortality rates by dividing the total estimated number of under-five deaths in a 12-month period by the total estimated number of births in the same period. These rates were then compared with the direct rates calculated from the gold-standard household mortality survey by calculating the ratios of the two rates.

Ethical clearance for the study, including the gold-standard mortality survey, was obtained from the Johns Hopkins School of Public Health's Institutional Review Board and the Malawi National Health and Science Research Committee.

## Results

**Figure 1:**
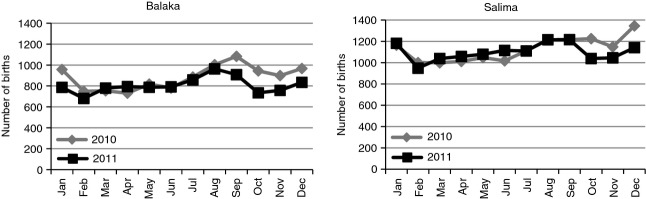
Distribution of health facility births by month and year in Balaka and Salima districts, Malawi, 2010 and 2011.

Figure 1 shows the distribution of health facility births by month in Balaka and Salima for calendar years 2010 and 2011. The monthly distribution of births is similar across years in each district, suggesting a good level of consistency in recording births. Numbers of births are presented in tabular form in Appendix S5.

**Figure 2:**
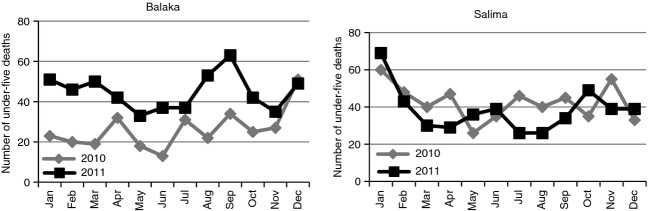
Distribution of health facility under-five deaths by month and year in Balaka and Salima districts, Malawi, 2010 and 2011.

Figure 2 shows under-five deaths reported in health facilities. There were important differences in the patterns of deaths between 2010 and 2011, especially in Balaka, where the number of under-five deaths reported was consistently higher in 2011 than in 2010. Reported deaths in Salima did not show a clear pattern by year. Numbers of deaths are presented in tabular form in Appendix S5.

In Balaka, the proportion of births in health facilities increased from 65.6% in 2005 to 76.2% in 2009 and then fell to 72.8% in 2010. In Salima, these proportions were 66.1% and 82.3% in 2005 and 2009, falling to 74.8% in 2010 (Table 2). In terms of deaths, based on the gold-standard survey, the proportion of deaths reported as occurring in health facilities in Balaka averaged 58.7%, with no clear pattern of change except for a sharp jump in 2011, whereas in Salima, the proportion held rather constant around a mean of 56.3% (Table 3).

**Table 2:** Proportional distribution of births by place and year of birth for Balaka and Salima districts, Malawi, 2005–2010 (DHS, 2010)

	Balaka				Salima			
		Per cent by place of birth				Per cent by place of birth		
Year of birth	Number of births	Health facility	Home	Other	Number of births	Health facility	Home	Other
2005	51	65.6	31.2	3.2	50	66.1	32.4	1.5
2006	98	65.5	28.1	6.4	108	63.2	35.5	1.3
2007	97	59.5	36.9	3.6	123	72.9	23.3	3.8
2008	110	63.3	32.3	4.3	110	70.1	24.6	5.3
2009	115	76.2	22.6	1.2	149	82.3	13.7	4.1
2010	61	72.8	24.1	3.1	53	74.8	13.0	12.2
Total	531	67.1	29.2	3.7	594	72.6	23.2	4.3

**Table 3:** Proportional distribution of under-five deaths by place and year of death for Balaka and Salima districts, 2005–2010 (gold-standard survey 2011–2012)

	Balaka				Salima			
		Per cent by place of death				Per cent by place of death		
Year of birth	Number of under-five deaths	Health facility	Home	Other	Number of under-five deaths	Health facility	Home	Other
2006	115	55.2	44.8	0.0	115	57.6	40.6	1.8
2007	168	59.8	38.8	1.4	160	56.9	42.5	0.6
2008	169	50.4	46.3	3.3	166	56.4	42.8	0.8
2009	177	57.9	38.9	3.2	212	50.4	48.1	1.5
2010	208	59.1	40.5	0.4	207	56.1	40.8	3.1
2011	145	70.3	28.6	1.1	139	62.6	34.1	3.3
Total	981	58.7	39.7	1.6	999	56.2	42.0	1.8

Table 4 presents the total number of births and under-five deaths reported in health facilities by district for five rolling 12-month periods beginning in January 2010, along with extrapolated number of births and under-five deaths to adjust for events outside health facilities. It also presents the expected number of births and under-five deaths and the ratios of the extrapolated numbers to the expected numbers. The ratios indicate that the extrapolated numbers of births are very close to the expected numbers of births, suggesting that estimates of numbers of births were accurate. This is not the case for under-five deaths, for which the extrapolated deaths are fewer than expected with ratios varying from 0.36 to 0.63 in Balaka and 0.52 to 0.57 in Salima.

**Table 4:** Health facility births and under-five deaths, extrapolated births and under-five deaths and expected births and deaths in Balaka and Salima districts, Malawi

Period	Estimated population*	Births from HMIS	Extrapolated births†	Expected births‡	Ratio reported births to expected births	Under-five deaths from HMIS	Extrapolated under-five deaths§	Expected under-five death‖	Ratio reported to expected under-five deaths
Balaka									
January 2010 – December 2010	338 430	10 570	14 191	13 635	1.04	315	487	1353	0.36
April 2010 – March 2011	341 103	10 356	13 903	13 743	1.01	400	618	1267	0.49
July 2010 – June 2011	343 776	10 399	13 961	13 850	1.01	449	694	1274	0.54
October 2010 – September 2011	346 448	10 157	13 636	13 958	0.98	515	796	1261	0.63
Salima									
January 2010 – December 2010	360 677	13 498	17 185	15 071	1.14	510	859	1626	0.53
April 2010 – March 2011	363 492	13 504	17 193	15 189	1.13	504	849	1642	0.52
July 2010 – June 2011	366 308	12 335	15 704	15 306	1.03	500	843	1521	0.55
October 2010 – September 2011	369 123	13 949	17 759	15 424	1.15	455	767	1337	0.57

* Based on projection from the Malawi National Statistical Office.

† Births are extrapolated using a proportion of birth in health facilities of 74.5% in Balaka and 78.5% in Salima.

‡ Expected births were obtained by multiplying the total population of by the estimated crude birth rate of 0.040 for Balaka and 0.042 for Salima computed from the gold-standard survey.

§ Under-five deaths are extrapolated using a proportion of under-five deaths in health facilities of 64.7% in Balaka and 59.3% in Salima.

‖ Expected under-five deaths were obtained by multiplying the expected births by the under-five mortality rate computed from the gold-standard survey.

Table 5 compares the extrapolated under-five mortality rates from health facility data with those from the gold-standard survey. The ratios indicate that the health facility extrapolation method captured only between 35% and 65% of under-five mortality as measured by the gold-standard survey in Balaka, and 46% and 50% in Salima. None of the mortality rates computed from adjusting health facility data fell within the 95% confidence interval of the mortality rate derived from the gold-standard survey. Reporting seems to have improved over time, especially in Balaka where the level of underestimation started at a high of 65% in 2010 but reduced gradually to 35% during the most recent 12-month period from October 2010 to September 2011.

**Table 5:** Adjusted estimates of under-five mortality rates (U5MR) from health facility data and corresponding under-five mortality rate from gold-standard survey with 95% confidence intervals

	Balaka				Salima			
		U5MR from gold-standard survey				U5MR from gold-standard survey		
Period	U5MR from adjusted Health Facility data	U5MR	95% CI	Ratio health facility to survey U5MR (%)	U5MR from adjusted Health Facility data	U5MR	95% CI	Ratio health facility data to RMM survey (%)
January 2010 – December 2010	34.3	99.2	82.8, 115.6	34.6	50.0	107.9	92.5, 123.2	46.4
April 2010 – March 2011	44.5	92.2	76.5, 107.9	48.2	49.4	108.1	93.1, 123.1	45.7
July 2010 – June 2011	49.7	92.0	76.2, 107.7	54.1	53.7	99.4	84.1, 114.6	54.0
October 2010 – September 2011	58.4	90.4	73.8, 106.9	64.6	43.2	86.7	73.2, 100.2	49.8

## Discussion

We tested the accuracy of annual under-five mortality rates generated from routine HMIS reporting of births and child deaths in health facilities in two districts in Malawi after adjusting for proportions of events occurring in facilities by comparing them at population level to rates produced by full birth history data collected through a high-quality household survey. The findings indicate that despite efforts to brief district and health facility staff on the importance of recording all child deaths, this method does not – at present – produce results that are sufficiently accurate to support sound decisions about progress in child survival. Rates generated from adjusted facility records were roughly half those generated for the same districts from a high-quality household survey.

The most obvious explanation for this finding is that staff in public and private health facilities do not record many of the deaths that occur on the HMIS forms. The current system used in Malawi does not support systematic recording of under-five deaths in all health facilities; only aggregate numbers of inpatient deaths are reported routinely. Deaths in paediatric wards are unlikely to be recorded at all, because reporting of these deaths is not required, and no appropriate register or form is in place to support such recording. Additionally, a review of the HMIS databases indicated that reporting from facilities is incomplete, with only district hospitals reporting inpatient deaths. Although the HMIS officers reported making frequent visits to health facilities to collect records of deaths by age group, there was still under-reporting of deaths within the HMIS system. The slight improvement over time observed in reporting in Balaka may be the result of proactive efforts by the HMIS officer from that district to visit maternity wards at health centres to compile reports of under-five deaths. The HMIS officer in Salima reported only deaths recorded in the district hospital. The findings reported here indicate that even when special efforts were made to increase the completeness of reporting, using health facility records to estimate all under-five deaths produced numbers and rates that were unacceptably low relative to the gold-standard survey.

A second potential explanation is that mothers interviewed in household surveys are not reporting accurately about where child deaths occur. If mothers are reporting that more deaths occurred in facilities than is actually the case – perhaps due to social desirability bias and not wanting to appear negligent –the proportion of deaths occurring in health facilities would be biased upwards. Thus, the extrapolated total number of deaths would be underestimated, resulting in an underestimation of extrapolated under-five mortality if we assume that there was no similar bias affecting reporting of births as occurring in health facilities.

The test of the method described here was based on health facility data collected using existing HMIS procedures, with the following exceptions. First, both district and health facility staff were informed about the purpose of the study. Second, modified reporting forms were introduced that included age groupings for deaths among children under 5 years of age and HMIS officers were provided with a monetary incentive to collect deaths by age. Third, an initial training of one day was conducted by the NSO in the use of these forms in February 2010. We did not introduce any other retraining or modification of the HMIS routine procedures for reporting births or deaths as a part of this assessment. There was, however, high turnover of HMIS officers posted at the district level during the study period, and new HMIS officers sometimes did not receive training on data collection from health facilities until a few months after their arrival. This occurred particularly in Balaka district and may explain the lower number of under-five deaths reported in 2010 in comparison with 2011. The selection of districts based on easy access to the study team may affect the generalisability of the findings. However, the easy access would have positively affected the results towards better agreement between the two methods. This was not the case, suggesting that this criterion did not have major positive effects on the findings.

The use of existing HMIS records of births and deaths in health facilities as a basis for estimating annual trends in under-five mortality is attractive because it can be implemented at low cost (assuming that survey data on the place of birth and, if applicable, death of children under 5 years of age are available) and reinforces the existing monitoring systems implemented by the Ministry of Health. However, this first assessment suggests that substantial efforts would be needed to change and maintain the reporting behaviours of staff at both health facilities and districts before this method could be used to generate data that were sufficiently sound to support decision-making. Unless efforts are made to modify the current HMIS to record deaths at all levels of health facilities systematically, the results are likely to continue to reflect high levels of underestimation. It is also important to keep in mind that computing child mortality by simply dividing under-five deaths by births during the same annual period will tend to underestimate the true under-five mortality if the number of births is increasing each year.

National and global policymakers must examine these results carefully to determine whether they are generalisable to other settings in sub-Saharan Africa and in other regions. First, this method is likely to produce unstable results in settings where lower proportions of births and deaths occur in health facilities. Second, Malawi has invested heavily in improving its HMIS system, even deploying a dedicated cadre of district HMIS officers to improve the quality of routine reports, and yet, there are still important flaws such as the absence of both age disaggregation for death reporting and requirements for death reporting for service settings other than inpatient wards. Third, further research is needed on the accuracy of mothers’ reports on place of death before the extrapolation of facility data to all births and deaths can be made with confidence.

Health information systems are an ideal data system to generate real-time data for monitoring programmes and decision-making (AbouZahr … Boerma 2005). However, our findings support those of others that have underscored the inability of the system to produce data of adequate quality to support programme and health system decision-making. The systems are generally set-up to collect data from health facilities and often focus on the technology of data collection rather than the use of the information produced for programme management (Gladwin 2003; Nyamtema 2010). Mutemwa states the problem clearly, suggesting that improvement in HMIS is not only limited in the adoption of an improved technology, but should also represent a ‘docking’ of this technology within the district health organisation system, aligning and reinforcing other sources of information within the district (Mutemwa 2006). Even the use of improved technology for data collection does not necessarily lead to improve data quality in terms of completeness and accuracy. Researchers in rural Tanzania report that the use of an electronic record system with careful double-data entry in sentinel health facilities did not resolve the completeness, accuracy and reliability issues that are common in HMIS data (Maokola *et al*. 2011). Those working to improve in HMISs must tackle the end goal of the system, which includes the use of data for management, monitoring and health system decision-making. This cannot be achieved without the involvement of the district health officers and HMIS officers in the regular review, analysis and interpretation of the data produced by the system. These data managers are uniquely capable of describing the data quality issues as a basis for resolving them (Braa *et al*. 2012).

Proponents of health systems strengthening should use these results as a basis for operational research to determine how, and under what conditions, health information systems in low- and middle-income countries can be strengthened to produce accurate and reliable measurements of under-five mortality, a core progress indicator. In the case of Malawi, expansion of the system to record all deaths at all levels, including first-level health facility, hospitals and private facilities, is a first and necessary step towards improving death recording in the HMIS system. Until this is performed, any calibration approach, including the one tested in this study, will continue to produce large underestimations of under-five mortality.

## Notes

Appendix S1. Demographic and health characteristics of districts in Malawi, with RMM districts highlighted.

Appendix S2. Malawi HMIS Health facility quarterly reporting form.

Appendix S3. Real-time monitoring health facility births and deaths monthly reporting form.

Appendix S4. Design, methods and data quality assessment results for the ‘gold-standard’ mortality survey.

Appendix S5. Raw numbers of births and deaths reported in health facilities in Balaka and Salima districts, by month, district and sex, 2010 and 2011.

## References

[b1] AbouZahr C, Boerma T (2005). Health information systems: the foundation of public health. Bulletin of the World Health Organization.

[b2] Braa J, Heywood A, Sahay S (2012). Improving quality and use of data through data-use workshops: Zanzibar, United Republic of Tanzania. Bulletin of the World Health Organization.

[b3] Commission on Information and Accountability for Women's and Children's Health (2011). Keeping Promises, Measuring Results.

[b4] Demographic and Health Surveys (2012). http://www.measuredhs.com.

[b5] Gething PW, Noor AM, Gikandi PW (2006). Improving imperfect data from health management information systems in Africa using space-time geostatistics. PLoS Medicine.

[b6] Gething PW, Atkinson PM, Noor AM, Gikandi PW, Hay SI, Nixon MS (2007a). A local space-time kriging approach applied to a national outpatient malaria dataset. Computers … Geosciences.

[b7] Gething PW, Noor AM, Goodman CA (2007b). Information for decision making from imperfect national data: tracking major changes in health care use in Kenya using geostatistics. BMC Medicine.

[b8] Gething PW, Noor AM, Gikandi PW (2008). Developing geostatistical space-time models to predict outpatient treatment burdens from incomplete national data. Geographical Analysis.

[b9] Gladwin J, Dixon RA, Wilson TD (2003). Implementing a new health management information system in Uganda. Health Policy and Planning.

[b10] Lohr SL (1999). Sampling: Design and Analysis.

[b11] Mahapatra P, Shibuya K, Lopez AD Civil registration systems and vital statistics: successes and missed opportunities. Lancet.

[b12] Malawi National Statistical Office (NSO) (2008).

[b13] Maokola W, Willey BA, Shirima K (2011). Enhancing the routine health information system in rural southern Tanzania: successes, challenges and lessons learned. Tropical Medicine and International Health.

[b14] Millennium Development Goals Indicators (2012). http://mdgs.un.org/unsd/mdg/Host.aspx?Content=Indicators/OfficialList.htm.

[b15] Multiple Indicator Cluster Surveys (2012). http://www.childinfor.org.

[b16] Murray CJ, Lopez AD, Barofsky JT, Bryson-Cahn C, Lozano R (2007). Estimating population cause-specific mortality fractions from in-hospital mortality: validation of a new method. PLoS Medicine.

[b17] Mutemwa RI (2006). HMIS and decision-making in Zambia: re-thinking information solutions for district health management in decentralized health systems. Health Policy and Planning.

[b18] National Statistical Office (NSO) and ICF Macro (2011). Malawi Demographic and Health Survey 2010.

[b19] Nyamtema AS (2010). Bridging the gaps in the Health Management Information System in the context of a changing health sector. BMC Medical Informatics and Decision Making.

[b20] Serwaa-Bonsu A, Herbst AJ, Reniers G (2010). First experiences in the implementation of biometric technology to link data from Health and Demographic Surveillance Systems with health facility data. Global Health Action.

[b21] Setel PW, Macfarlane SB, Szreter S (2007). A scandal of invisibility: making everyone count by counting everyone. Lancet.

[b22] STATcompiler (2012). http://www.statcompiler.com/.

