# Supplementary material for: Monitoring child survival in ‘real time’ using routine health facility records: results from Malawi
Source: Trop Med Int Health. 2013 Aug 1;18(10):1231–9. doi: 10.1111/tmi.12167 (PMC3787785; doi:10.1111/tmi.12167)
Supplement: Appendix S1. — Demographic and health characteristics of districts in Malawi, with RMM districts highlighted. [file tmi0018-1231-SD1.pdf]

APPENDICES:

1. Demographic and health characteristics of districts in Malawi, with RMM districts highlighted.
2. Malawi HMIS Health facility quarterly reporting from
3. Real-time monitoring health facility births and deaths monthly reporting form
4. Design, methods and data quality assessment results for the “gold-standard” mortality survey.
5. Raw numbers of births and deaths reported in health facilities in Balaka and Salima districts, by month, district and sex, 2010 and 2011.

APPENDIX 1: Demographic and health characteristics of districts in Malawi, with RMM districts highlighted

Region and districts	Population[1]	U5MR[2]	TFR[2]
**NORTH**			
Chitipa	174,693	99	6.8
Karonga	258,074	83	5.6
Mzimba	787,445	115	5.3
Nkhata Bay	202,388	81	4.5
Rumphi	160,298	99	6.2
**CENTRAL**			
Dedza	669,511	160	7
Dowa	526,604	139	6.2
Kasungu	671,391	132	6.1
Lilongwe	2,033,049	166	6.8
Mchinji	456,314	133	5.7
Nkhotakota	310,909	118	6.2
Ntcheu	511,184	149	5.6
Salima	340,327	144	7.1
**SOUTH**			
**Balaka**	**316,748**	**160**	**6.3**
Blantyre	1,203,398	121	4.6
Chikwawa	477,534	147	6.9
Chiradzulu	309,012	159	5.6
Machinga	447,243	117	6.9
Mangochi	802,568	150	8
Mulanje	575,014	107	4.5
Mwanza	184,862	137	6
Nsanje	245,927	171	6.7
Phalombe	322,409	161	6.9
Thyolo	611,424	123	6.2
Zomba	747,620	138	5.5

Notes:
[1] 2008 projection from National Statistical Office, Malawi
[2] From MICS 2006

Appendix 2: Malawi Health Management Information System quarterly reporting form

Fiscal Year: HMIS- 15

___________________________ Hospital/ Health Centre

Health Management Information

Quarterly Report

Facility Code:

Indic No.	Data Elements (DE)	Month	Month	Month	Quarterly Total
	**Maternal Services**				
39	Number of pregnant women starting antenatal care during their first trimester				
40	Total number of new antenatal attendees				
40	Total antenatal visits				
41	Number of deliveries				
42	Number of women with obstetric complications treated at obstetric care facility				
43	Number of caesarean sections				
44	Total number of live births				
44	Number of babies born with weight less than 2500g				
45	Number of abortion complications treated				
46	Number of eclampsia cases treated				
47	Number of Postpartum haemorrhage (PPH) cases treated				
48	Number of sepsis cases treated				
49	Number of pregnant women treated for severe anaemia				
51	Number of newborn treated for complications				
52	Number of postpartum care within 2 weeks of delivery				
	**Family Planning**				
53a	Number of persons receiving 3 months supply of condoms				
b	Number of persons receiving 3 months supply of oral pills				
c	Number of persons receiving Depo-Provera				
d	Number of persons receiving Norplant				
e	Number of persons receiving IUCD				
f	Number of persons receiving sterilisation method of F/P				
	**Child Health**				
55	Number of fully immunised under 1 children				
56	Number of under one children given BCG				
56	Number of under one children given Pentavalent 111				
56	Number of under one children given Polio 111				
56	Number of under one children given Measles 1st doses at 9 months				
57	Number of vitamin A doses given to 6-59 months population				
62	Number of under weight in under fives attending clinic				
	**Attendance**				

Fiscal Year: Facility code:

Indic No.	Data Elements (DE)	Month	Month	Month	Quarterly Total
30	Number of 15-49 years receiving volunteer and confidential testing and serostatus result				
31	Number of 15-49 age group tested HIV positive				
32	Number of HIV positive persons receiving ARV treatment				
34	Number of pregnant women receiving VCT and serostatus result				
35	Number of pregnant women tested HIV positive				
36	Number of HIV positive women treated for PMTCT				
62	Total number of children attending under-five clinic				
103	Number of OPD attendance				
	**Tuberculosis**				
65	Number of confirmed TB new cases				
66	Number of smear negative and extra-pulmonary cases completed treatment				
67	Number of new sputum positive cases proved smear negative at the end of treatment				
	**Supplies**				
23	Was there any stock outs of SP for more than a week at a time? (Y/N)				
23	Was there any stock outs of ORS for more than a week at a time? (Y/N)				
23	Was there any stock outs of cotrimoxazole for more than a week at a time? (Y/N)				
23	Was there any stock outs of SP, ORS and cotrimoxazole for more than a week at a time? (Y/N)				
24	Number of functioning ambulances				
76	Number of insecticide treated nets distributed				
	**Community Health Activities**				
25	Number of households with access to safe drinking water				
26	Number of households with at least a sanplat latrine				
38	Number of HBC patients followed-up and provided treatment				
	**Human Resources Currently at Work**				
7	Clinical officers				
	Doctors				
	Dental Surgeon				
	Dermatologist				
	Medical Officer				
	Obs/gynaecologist				
	Ophthalmologist				
	Paediatrician				
	Pathologist				
	Physician				
	Surgeon				

Fiscal Year: Facility code:

Indic No.	Data Elements (DE)	Month	Month	Month	Quarterly Total
	Environmental Health Officer				
	Health Surveillance Assistant				
	Medical Assistant				
	Nurses				
	Registered				
	Enrolled/Midwife				
	Community				
	Pharmacist				
	Physiotherapist				
	Radiologist				
	Technicians				
	Laboratory				
	Pharmacy				
	Radiograph				
	All other positions				
	Total personnel currently at work				
	**Finance**				
56	Total income from cost sharing				
	**Physical Facilities**				
17	Do you have functioning water supply system? (Y/N)				
17	Do you have functioning electricity (Y/N)				
17	Do you have functioning communication system? (Y/N)				
17	Do you have functioning water supply, electricity and communication system? (Y/N)				
	**Management and Supervision**				
13	Is the health centre committee functional? (Y/N)				
15	Were you supervised by DHMT members using the integrated supervision checklist? (N/Y)				
	**New Cases (OPD plus Inpatient)**				
27	Sexually transmitted infections – new cases				
29	Syphilis in pregnant				
31	HIV confirmed positive (15 - 49) new cases				
37	Opportunistic infections – new cases				
58	Acute Respiratory Infections – new cases (under-5)				
60	Diarrhoea non-bloody – new cases (under-5)				
64	Malnutrition – new cases (under-5)				
69	Malaria – new cases (under-5)				
70	Malaria – new cases (5 & above)				
78	Neonatal tetanus – confirmed new cases				
79	Cholera – confirmed new cases				
81	Measles – confirmed new cases				
82	Acute Flaccid Paralysis – confirmed new cases				
83	Ebola – confirmed new cases				
84	Meningococcal meningitis – confirmed new cases				
85	Plague – confirmed new cases				

Fiscal Year: Facility code:

Indic No.	Data Elements (DE)	Month	Month	Month	Quarterly Total
86	Rabies – confirmed new cases				
87	Yellow fever – confirmed new cases				
88	Dysentery – new cases				
90	Eye infections – new cases				
91	Ear infection – new cases				
92	Skin infections – new cases				
93	Oral conditions (including dental decay) – new cases				
94	Schistosomiasis – new cases				
95	Leprosy – new cases				
96	Common injuries and wounds (except RTA)				
98	Number of road traffic accidents				
	**Admissions**				
20a	Bed capacity				
20b	Total number of admissions (including maternity)				
20c	Total number of discharges				
20d	Total number of inpatient days				
	**Inpatient Deaths (including Maternity Deaths)**				
102	Total number of inpatient deaths from all causes (excluding maternity)				
50	Number of direct obstetric deaths in the facility				
59	Acute Respiratory Infections – inpatient deaths (under-5)				
61	Diarrhoea non-bloody (under-5) – inpatient deaths				
64	Malnutrition – inpatient deaths (under-5)				
68	TB – inpatient deaths				
69	Malaria – inpatient deaths (under-5)				
74	Malaria – inpatient deaths (5 & above)				
80	Cholera – Inpatient deaths				
89	Dysentery – Inpatient deaths				
98	Number of road accidents – Inpatient deaths				

Report prepared by Signature Date

Report verified by Signature Date

Report approved by Signature Date

Notes: 1) HMIS – 13 has to be completed before transcribing data onto this form.

2) Management team has to analyse data and provide feed back to its staff before sending report to DHO.

3) This Quarterly report is due on 15th October, January, April and July.

APPENDIX 3: Real-time monitoring health facility births and deaths monthly reporting form

Evaluation of Maternal, Newborn and Child Health Rapid Scale-Up in Malawi
Real-time Mortality Monitoring
Monthly Health Facility Births and Deaths Monitoring

Reporting period (dd /mm/20YY)	_ _/_ _/ TO _ _/_ _/ _ _ _ _		
District	________________________		
Health Facility Name	________________________		
Date (dd /mm/20YY)	_ _/_ _/ _ _ _ _		
Total number of deliveries	_ _ _		
Total number of live births	Female<br>_ _ _	Male<br>_ _ _	Total<br>_ _ _
Total number deaths in the health facility by age and sex	Female	Male	Total
Less than 28 days<br>*(less than 1 month old)*	_ _	_ _	_ _
28 days to 11 months<br>*(1 month to less than 1 year old)*	_ _	_ _	_ _
12 months to 59 months<br>*(1 year to less than 5 years old)*	_ _	_ _	_ _
60 months or more<br>*(5 years old or more)*	_ _	_ _	_ _
Number of maternal deaths<br>*(death of a pregnant woman, death during delivery or within 42 days after delivery)*	_ _		

Name of data recorder:

<u>APPENDIX 4</u>: Design and methods for the "gold-standard" mortality survey

The aim of the RRT project was to provide rigorous validations of promising approaches, as a basis for making decisions about which methods justify broader application within Malawi and in other, similar settings. The best available method for measuring under-five mortality was the collection of full birth histories from women of reproductive age in high-quality household surveys conducted using the methods developed under the Demographic and Health Survey program supported by USAID. IIP-JHU worked in collaboration with the Malawi NSO to design and carry out such a survey in two RRT districts in Malawi. The survey was designed to serve as the gold standard against which the data generated through RRT methods could be validated, and thus, included rigorous quality control measures.

B.1. Objective

The main objective of the household mortality survey was to obtain the data needed to validate the three RRT approaches implemented in two districts in Malawi. More specifically the survey was designed to:

1) Estimate childhood mortality in the districts of Balaka and Salima, based on women's report of their full birth history; and
2) Support validation of the RRT methods implemented in Balaka and Salima.

B.2. Methods

B.2.1. Sample size

The survey collected data from 24,000 households distributed equally between the two RRT districts. Section 8.1.2 above provides details of the sample size calculation.

B.2.2. Sampling frame and sample selection

The 2008 Population Census frame was used to select the primary sampling units or enumeration areas (EA) for the survey, with probability proportionate to size. The sampling was stratified by district.

The plan was to select 35 households per EA, making a total of 686 EAs, equally divided across the two districts. Thus, 343 EAs were selected in each district. In Balaka, the total number of EAs in the entire district was 294. However, there were large variations in the size of EAs, ranging from 21 to 650 households with an average number of 253 households. To reach the 343 EAs needed in the sample, large EAs with more than 330 households were divided into two or three EAs, and all resulting EAs in the district were included in the sample (now a census of EAs). All households within each EA were listed and a sample of 35 households was selected using a systematic random sampling procedure. Thus, in Balaka, only a single stage sample of households was conducted.

In Salima, the total number of EAs is 435. Two-stage sampling was used, with EA selection at first stage and household selection at a second stage. The primary stage selection was conducted using a systematic random sampling procedure with probability proportionate to size in terms of households. The size of EAs varied from 15 to 780 households. During the sample selection, some very large EAs were selected twice; one EA was selected three times and another four times. These EAs were divided into the number of times they were selected, and each segment used a separate cluster. In addition, very small EAs were combined with neighboring EAs before the random selection was completed. All households in each selected EAs were listed and a random selection of 35 households occurred using systematic random sampling procedures.

Given that the same number of households was selected in each cluster, the probability of selection of a household was not the same for every home included in the sample. Thus, sampling weights were computed and used during analysis to ensure the representativeness of the results.

B.2.3. Questionnaires

These authors developed a structured, pre-coded questionnaire adapted from the standard DHS tool. The household form collected basic information about each household member as well as gathering information about access to water and sanitation facilities, roof, floor and wall construction materials, number of sleeping rooms, possession of land and livestock ownership and household ownership of assets such as mobile phones, bicycles, radios, and others items. GPS coordinates were collected for each household.

The “individual woman” form was administered to all women ages 15-49 living within the household. Women were asked to provide age, marital status, and educational level. They were asked a summary list of questions on whether they had ever given birth, and if so, how many of their children were still alive at the time of the interview. If a woman reported having children, a full birth history module was administered that included detailed questions about each child.

All questions were written in both English and the local language of Chichewa to assist the interviewers, but interviews were conducted in Chichewa most often. The tool was pre-tested during interviewer field practice sessions in rural villages near Zomba in October 2011. To ensure the highest possible survey quality, the questionnaire was kept short and administration took 15 to 30 minutes.

B.2.4. Recruitment and training of fieldworkers

The Human Resources Department of the NSO assisted by technical staff (survey managers) led the process of recruiting field staff. This involved advertising, short listing, interviewing, reviewing candidates’ performance in the written skill test, and selecting successful candidates.

In August 2011, advertisements for the post of field staff was made in local newspapers, calling for would be candidates to fill the post of survey enumerators. The required credentials included possession of a Malawi School Certificate of Education (MSCE) with credits in Mathematics and English; an

equivalent of the British O Level certificate, and experience in household surveys and specifically the Malawi Demographic and Health Survey (MDHS). The advertisements were maintained for three weeks.

Short listing commenced immediately upon receipt of applications and potential interviewees were selected based on the standard criteria listed above. The interview was primarily skill-based with each candidate presented with a written case study and asked to complete the RRT survey questionnaire in writing. The completed questionnaires were marked by survey managers, with special attention to whether the candidate demonstrated a correct understanding of the questionnaire. Successful candidates were called for training one week later.

Training commenced in 10 October 2011 and continued for eight days, including two days of supervised field practice. Training methods included classroom training on each questionnaire module, mock interviews, plenary discussions, and supervised field practice. The training explained how to complete the household questionnaire, including the information panel, the listing of household members and their characteristics and information on water and sanitation. The individual woman's questionnaire included questions pertaining to her characteristics and the summary of full birth histories. Other sessions focused on how to conduct a successful interview, the roles, and responsibilities of field staff, mapping using a global positioning system (GPS), sampling, household listing, and checking and editing competed questionnaires. Field practice sessions focused on improving field staff capability to locate households, conduct interviews, and edit questionnaires. Plenary sessions were held at the end of each field practice exercise to discuss the experiences of field staff and exchange knowledge as well as to fine-tune weak areas observed by field practice supervisors. A final test was conducted and used to select the best-performing trainees, identify supervisors, and for forming well-balanced field teams.

B.2.5. Fieldwork

Fieldwork started on 24 October 2011 and ended on 17 February 2012. A survey coordinator was appointed for each district, along with eight survey teams composed of one supervisor, five enumerators and a driver, with 16 survey teams. At district level, the survey coordinator ensured that all responsible district authorities (District commissioner, Officer in Charge at Police, Traditional leaders) were notified about the survey and its entire organization (the teams, tasks, location, and timing of data collection).

The survey coordinators were the link between the field staff on the ground and the survey coordinating personnel at the NSO headquarters. Each survey coordinator was responsible for logistical and technical backstopping to the teams. This involved ensuring availability of logistical supplies such as fuel for the vehicles, questionnaires, listing forms, maps, chalk, and every other equipment and consumables required during fieldwork, throughout the survey period.

At the team level, a supervisor was responsible for coordination of field workers' daily assignments and the listing, interviewing, and editing of questionnaires. The teams began in each EA by first listing all the households. Then the team supervisor randomly sampled 35 households and the interviews commenced immediately.

The team supervisor ensured that a daily schedule was prepared, outlining the tasks to be done, the location where the task was to be performed, and the team responsible for the task. The team leader also ensured that all teams had the necessary supplies. At the end of each field day, interviewers edited questionnaires before submitting them to their team leader, who also reviewed each questionnaire, conducted callbacks, and re-interviewed as needed.

Four senior survey supervisors from NSO visited field teams on a rotating basis throughout the survey period, conducting on-the-spot supervision. Key areas of focus during these visits were checking whether mapping was correct, observing how interviews were conducted, assessing data quality, and providing on-the-spot corrections. Upon their return to NSO, any serious anomalies were discussed at the central coordinating office with the survey senior management team. Decisions were communicated to responsible field personnel immediately. Periodic supervision reports at all levels of survey coordination and implementation were produced and provided further input for improving survey management.

B.2.6. Quality Control

A number of quality control mechanisms were put in place. During fieldwork, team leaders were required to observe at least one interview from each interviewer in a cluster. They were also required to conduct a re-interview in one household for each interviewer.

After completion of the interviews, the team leader checked all the completed forms for inconsistencies and signed the edited questionnaires that were satisfactory. Questionnaires with inconsistencies were sent back to the interviewers for correction. Guidelines for questionnaire editing were detailed in the Supervisors' Manual for the survey. At district level, a field coordinator spot-checked all completed questionnaires and initialed them for onward transfer to NSO. When the forms arrived at the NSO, four office editors scrutinized the questionnaires for any inconsistencies before sending them on to the data entry unit at NSO.

B.2.6. Data entry and processing

All completed data forms were packed in A3 envelopes with a dispatch form, a listing form, and the Interviewer and Supervisor assignment sheets. This packet was sent to the NSO for data processing. Upon arrival at the NSO, forms were registered in a questionnaire registry inventory file and spreadsheet. Four office editors were trained to check the questionnaires for any inconsistencies using prescribed data editing guidelines.

Once the editing was completed, questionnaires from each cluster were passed on to the assigned data entry clerk. A CSPro 4.1 data application program was developed that allowed for double data entry. Sixteen data entry clerks were trained on data processing procedures. Once data for a cluster were entered, the forms were passed to a second data entry clerk for double verification to eliminate entry errors. The two datasets were then checked and reconciled.

Completed data sets were then transferred to STATA for further cleaning. All data collected on GPS were copied by the appropriate software and stored in a separate file. All data analyses were conducted in STATA.

B.2.7. Survey results

The survey successfully reached a household response rate of 98.5%, 99.1% in Balaka and 97.9% in Salima. Eligible women response rates were estimated at 96.3% (95.7% in Balaka and 97.9% in Salima). These rates are comparable to the 2010 DHS rates.

Table B1: Results of households and women's interviews

Result	District		
	**Balaka**	**Salima**	**Total**
Sample households	12005	12005	24010
Interviewed households	11,899	11,749	23,648
**Households response rate**	**99.1**	**97.9**	**98.5**
Eligible women in interviewed households	10,787	11,117	21,904
Women interviewed	10,322	10,763	21,085
**Women's response rate**	**95.7**	**96.8**	**96.3**

B.2.7. Household population in Balaka and Salima

Table B2: Percent distribution of the de facto household population by five-year age groups, according to sex and district

Age group	Total			Balaka			Salima		
	Males	Females	Total	Males	Females	Total	Males	Females	Total
0-4	18.6	18.0	18.3	18.2	18.0	18.1	19.0	17.9	18.5
5-9	17.8	16.9	17.3	18.0	16.8	17.3	17.5	17.0	17.3
10-14	14.6	14.0	14.3	14.7	14.0	14.3	14.5	14.1	14.3
15-19	10.3	9.4	9.8	10.4	9.2	9.8	10.1	9.7	9.9
20-24	7.2	8.5	7.8	7.3	8.4	7.9	7.1	8.5	7.8
25-29	7.0	7.7	7.3	6.8	7.6	7.2	7.2	7.8	7.5
30-34	5.6	5.5	5.6	5.5	5.5	5.5	5.6	5.6	5.6
35-39	4.9	4.2	4.5	4.7	4.0	4.3	5.1	4.5	4.8
40-44	3.2	2.6	2.9	3.0	2.7	2.9	3.4	2.5	2.9
45-49	2.4	2.1	2.3	2.4	2.2	2.3	2.5	2.0	2.3
50-54	1.9	3.1	2.5	2.0	3.3	2.7	1.9	2.9	2.4
55-59	1.7	2.0	1.9	1.6	2.1	1.9	1.7	2.0	1.8
60-64	1.9	2.0	1.9	2.1	2.2	2.1	1.6	1.7	1.7
65-69	1.1	1.3	1.2	1.1	1.2	1.2	1.0	1.3	1.2
70-74	0.8	1.1	1.0	0.9	1.1	1.0	0.8	1.0	0.9
75-79	0.5	0.7	0.6	0.5	0.8	0.7	0.5	0.6	0.6
80+	0.7	0.9	0.8	0.8	0.9	0.9	0.5	0.8	0.7
Missing	0.0	0.0	0.0	0.0	0.0	0.0	0.0	0.0	0.0
Total	100.0	100.0	100.0	100.0	100.0	100.0	100.0	100.0	100.0
Number	52,039	54,648	106,687	25,388	27,257	52,645	26,701	27,341	54,042

Figure B1: Population age pyramid for Balaka and Salima

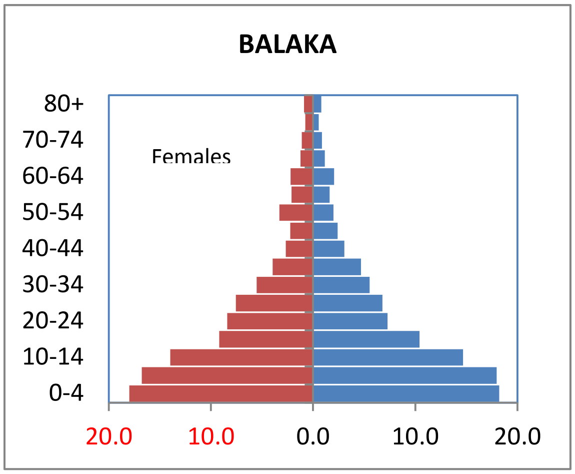

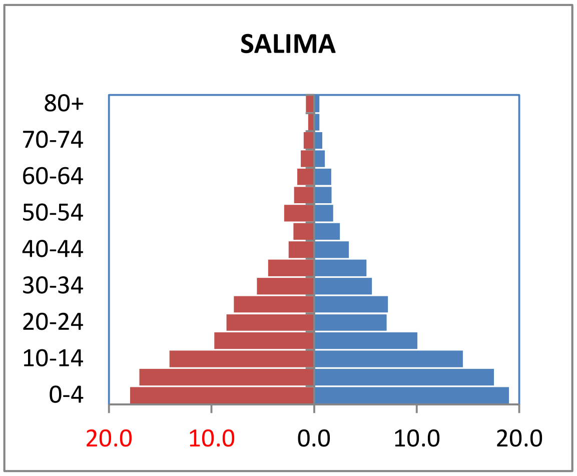

B.2.7. Data quality assessment

This team conducted preliminary internal quality assessment of the data by examining the age and date of birth. A more extensive quality assessment is currently ongoing.

a) Transfer of eligible females outside eligibility age range

Figure B1 shows the age distribution of females aged 9 to 54 years in the sampled households. In both districts, there are drops in the number of female household residents between the ages of 12 and 15, and increases at ages 50-52, suggesting some transference of eligible women outside the age eligibility range. This phenomenon is characteristic of household surveys where interviewers are required to identify women of reproductive age in the households for further interview. Figure B1 compare the age distribution of females to that from the DHS 2010 data. The age distribution is very similar.

Figure B1: Distribution of household females by age and by districts (only age 9-54 years are shown)

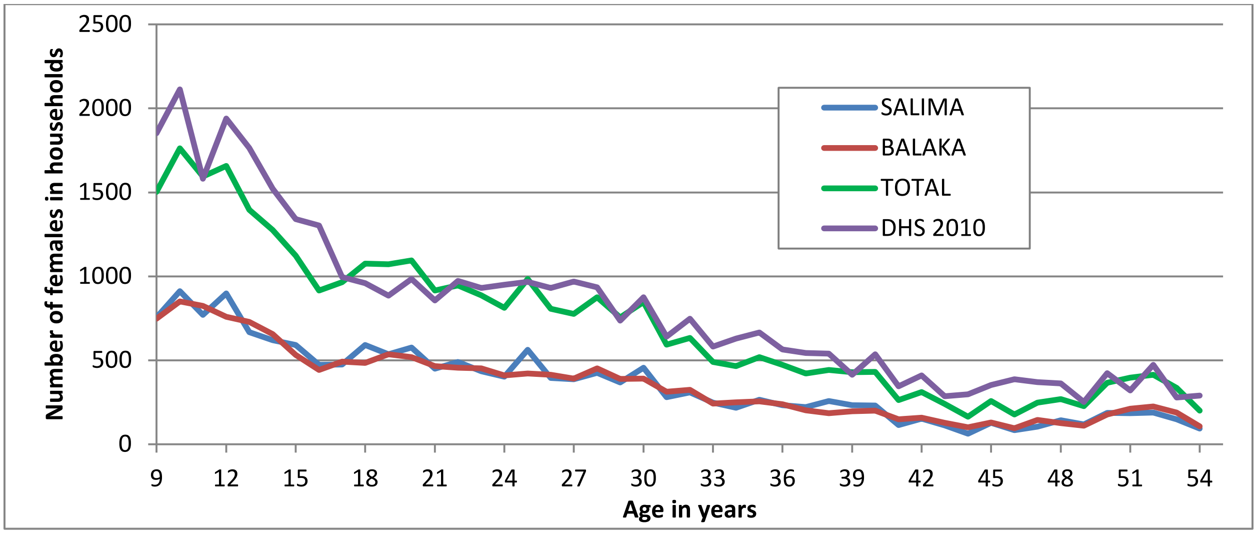

b) Births by month and year

Figures B2 and B3 show the distribution of births by month and year from 2001 to 2011 for Balaka and Salima districts, respectively. The patterns look generally similar across years. In Salima, there is a consistent reduction in the number of births reported across years. In both districts, the bottom curve (in red) indicates the patterns in 2002 when the country experienced a severe famine. The patterns of distribution of births in Balaka and Salima are also similar to the nationwide patterns observed in data from the DHS 2010 (figure B4).

Figure B2: Distribution of births by month and year of birth in Balaka

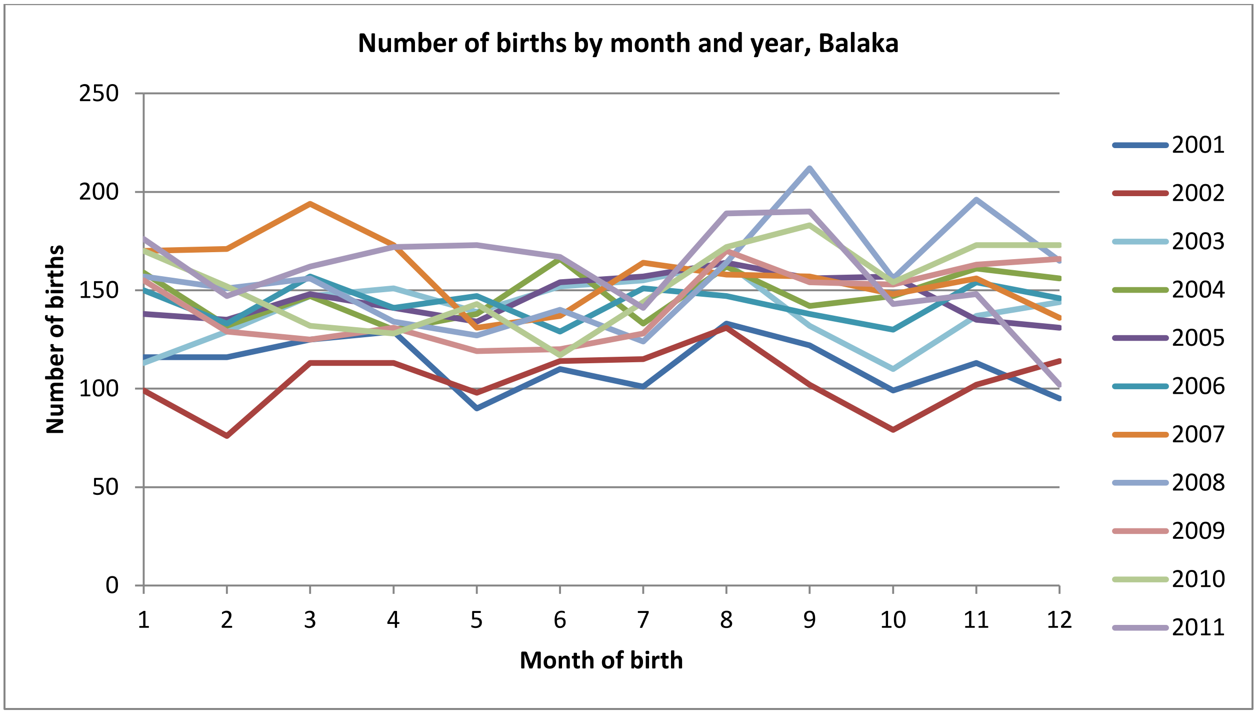

Figure B3: Distribution of births by month and year of birth in Salima

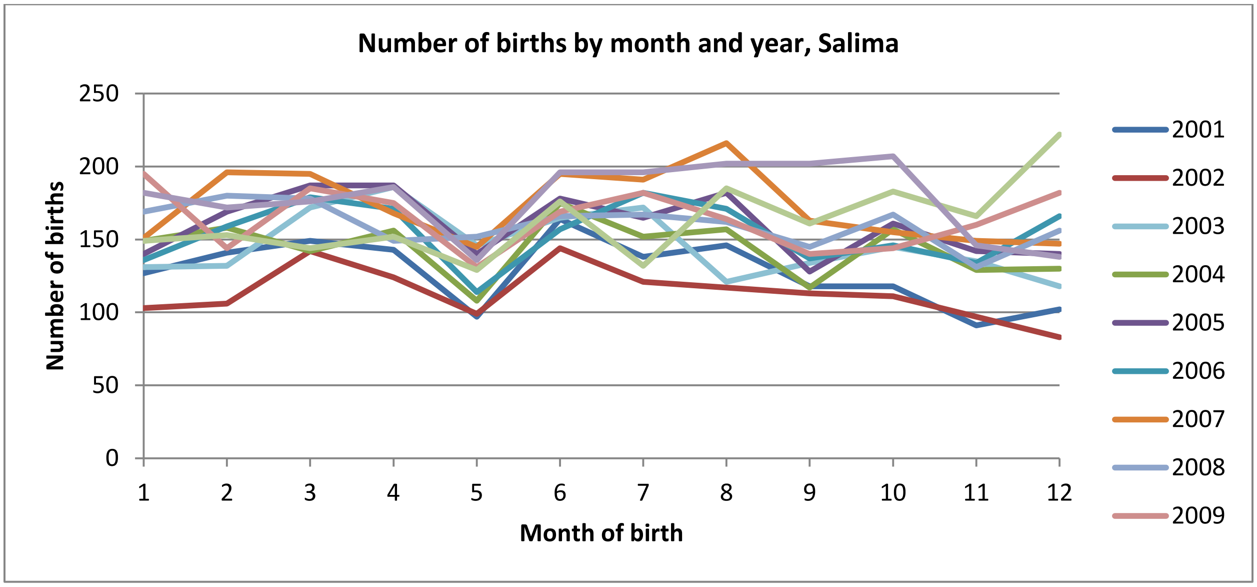

Figure B4: Distribution of births by month and year of birth Malawi, DHS 2010

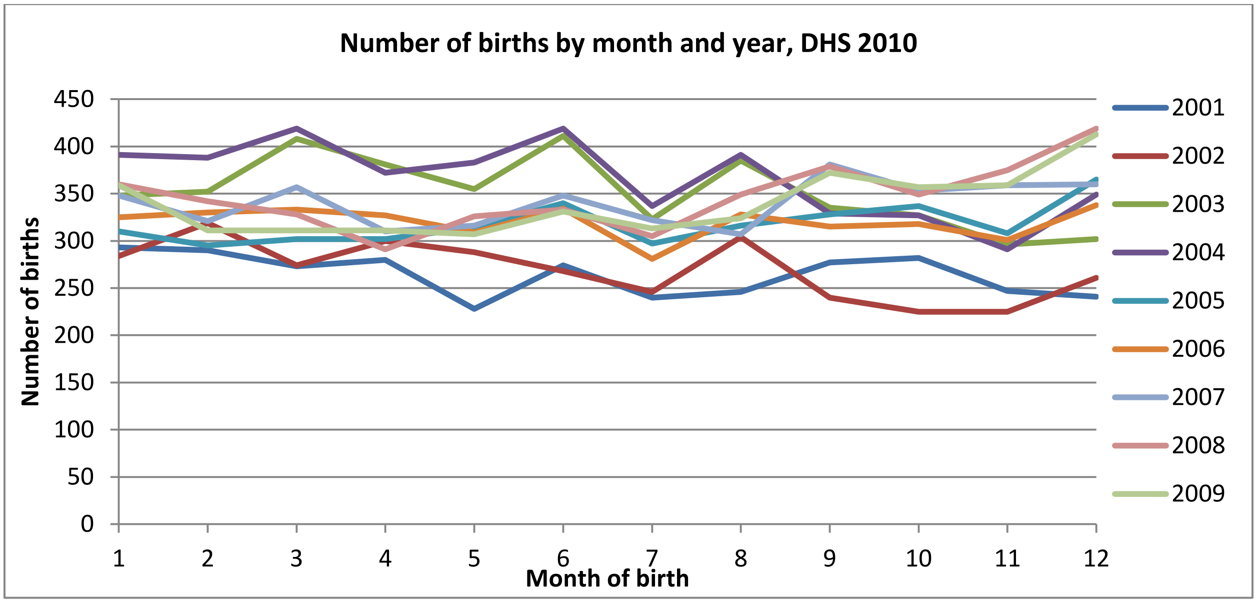

c) Sex ratio at birth

The sex ratio at birth in normal populations is generally between 102 to 107 males per 100 females. Large departures from this range may suggest differential omission of births by sex. Figures B5, B6 and B7 show sex ratios at delivery for all births, living children, and children who died. Sex ratios at delivery for all births and for living children do not indicate any severe irregularities and compare fairly well with the similar sex ratios from DHS 2010. However, sex ratios for children who have died show a large dip in 1993 and prominent peaks in 1995/97. These may be indicative of specific contextual factors that will need further documentation. These patterns are reflected in the 2010 DHS data although the trends in the DHS 2010 data are not as extreme.

d) Age at death

Errors in age declaration can affect mortality estimation. Figures B7 and B8 show the distribution of neonatal deaths by age in days and the per month distribution of children who died between the ages of one and 24 months by age at death. The observed distributions are typical of empirical surveys and the tendency for deaths to "heap" at certain ages. For age at death for neonates, these data show heaping at ages one, seven, and 14 days. For children who died between the ages of one and 23 months, the distribution shows stronger heaping at age 12 months. This particular pattern would lead to an under-estimation of the infant mortality rate and an over-estimation of the child mortality rate. The under-five mortality rate will not be affected.

Figure B5: Sex Ratio at birth for births since 1990

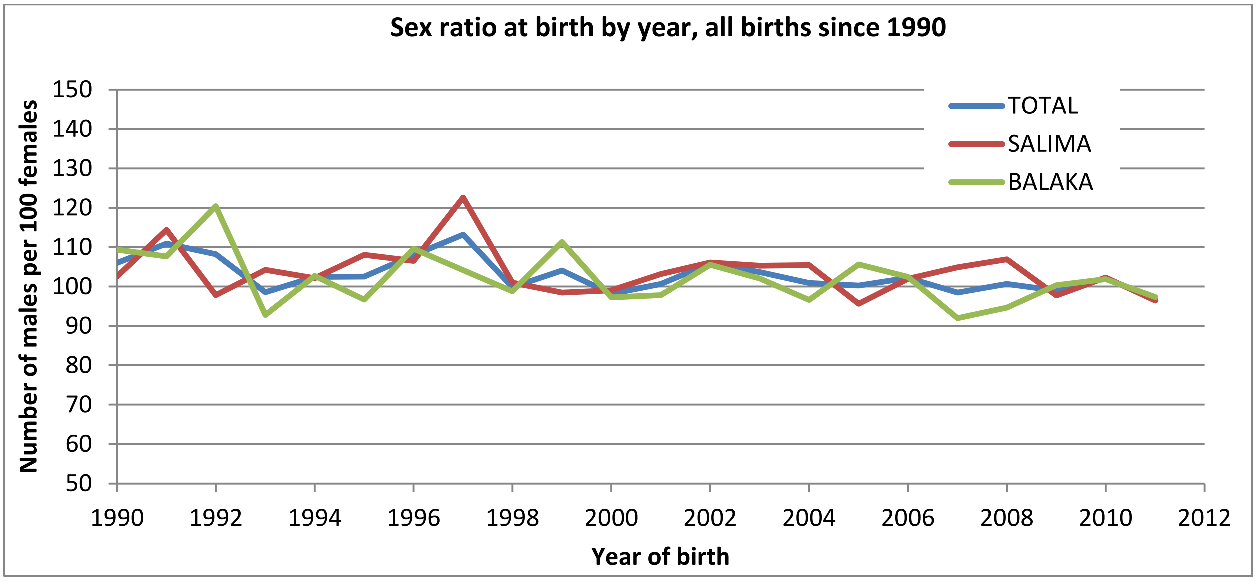

Figure B6: Sex-ratio at birth for living children

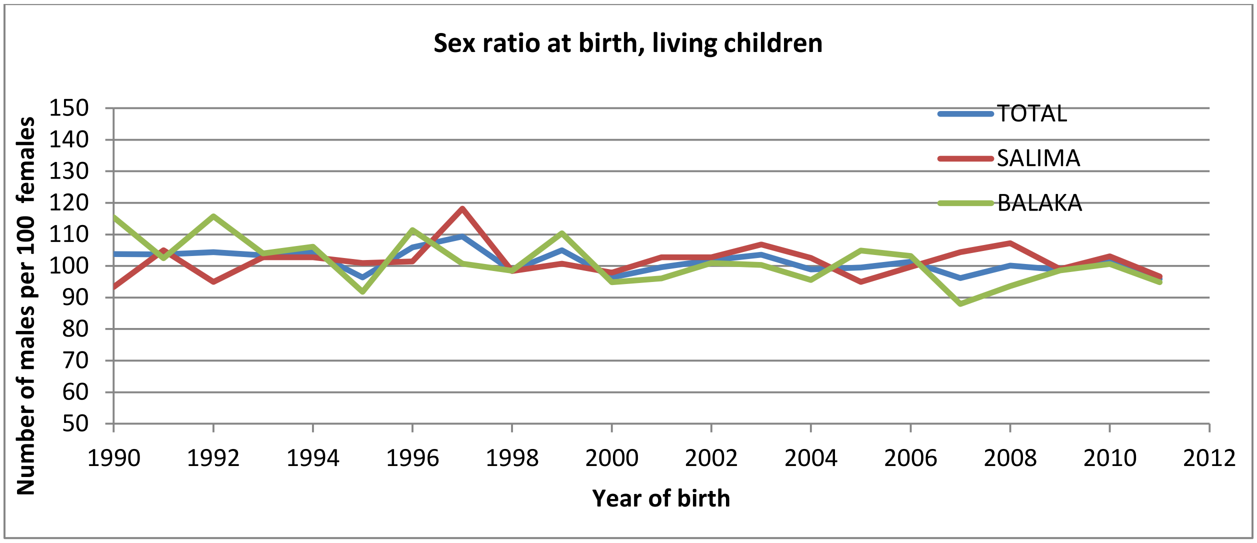

Figure B7: Sex-ratio at birth for children who have died

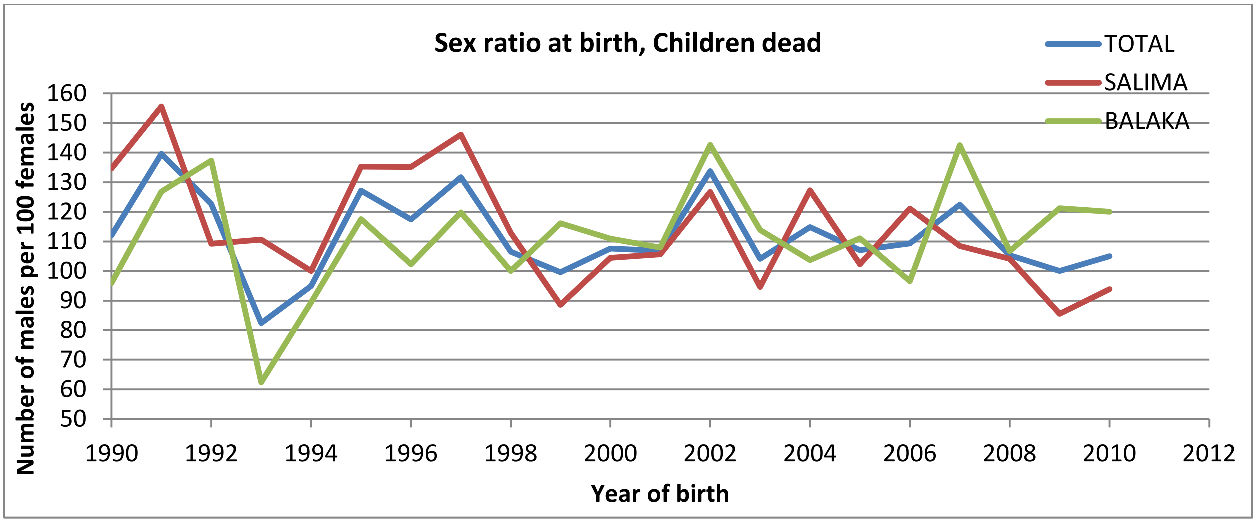

Figure B8: Sex-ratio at birth for all, living and dead children, DHS 2010

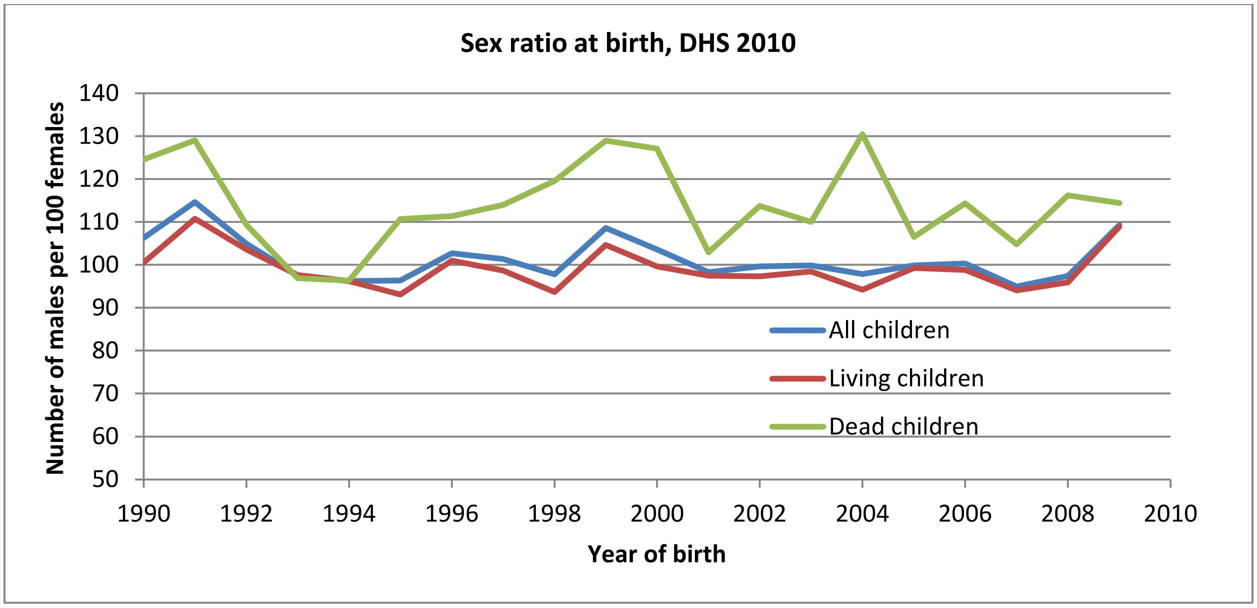

Figure B7: Distribution of neonatal deaths by age at death in days (births since 1990)

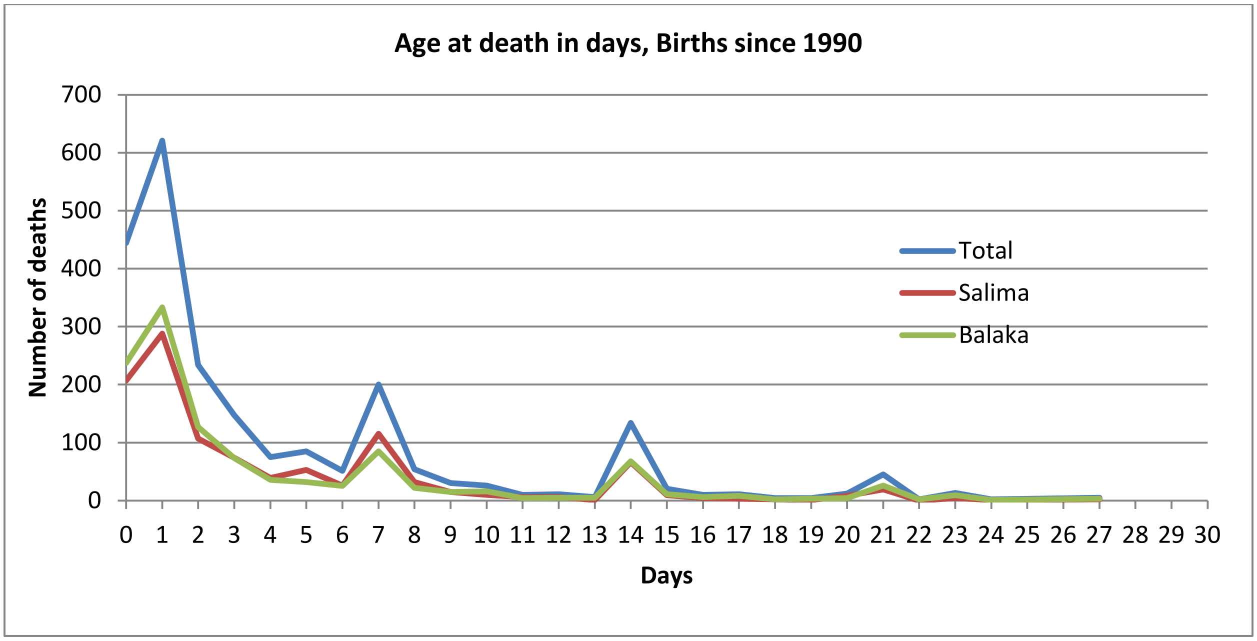

Figure B8: Distribution of children who died between ages of 1 month and 24 months by age at death in month (births since 1990)

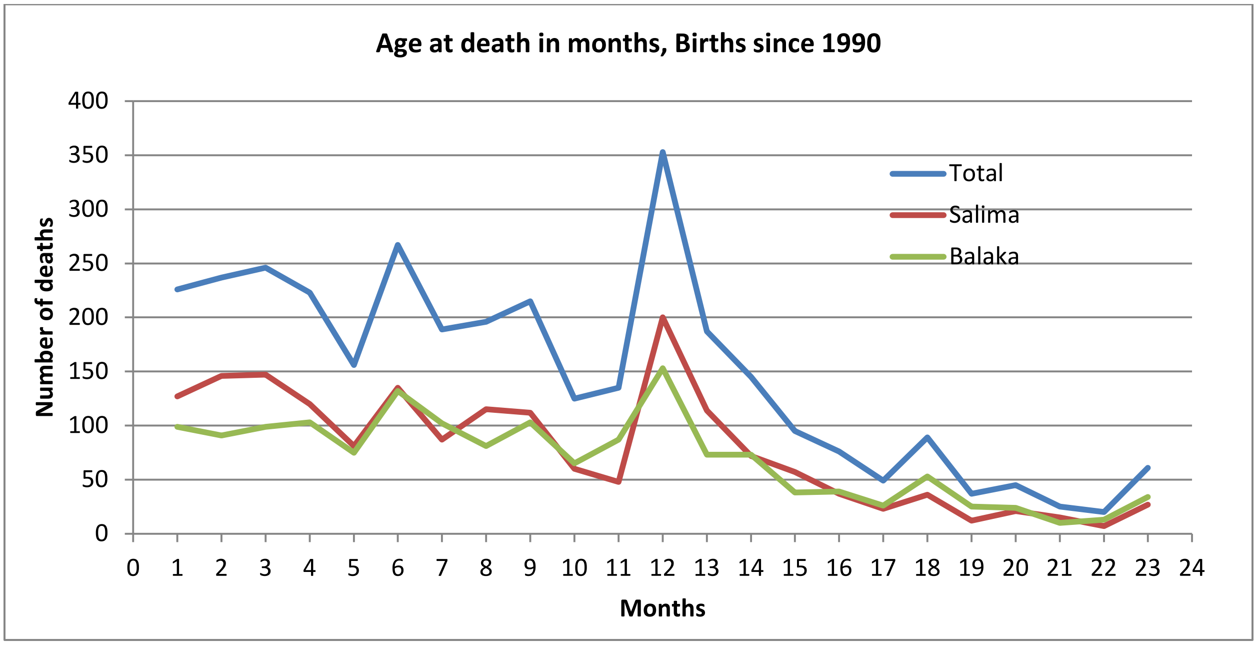

e) Age patterns of deaths: ratio of early neonatal to neonatal deaths and neonatal deaths to infant deaths

Table B3 presents the ratios of early neonatal to neonatal deaths and the ratio of neonatal to under-five deaths for the RRT and the 2010 DHS surveys. The ratio of early neonatal to neonatal deaths is generally within the range of 0.70 to 0.80. The ratios presented for the RRT survey in either Balaka or Salima are in the same range. However, compared to the DHS 2010, there is difference between the ratio in Salima. The DHS ratio is on the lower side suggesting an under-reporting of early neonatal deaths in the DHS.

The ratio of neonatal to under-five death is in the range of 0.30 in Balaka or Salima and is comparable to the DHS ratio.

Table B3: Ratio of early neonatal to neonatal deaths and ratio of neonatal to under-five deaths from the RRT survey and the 2010 DHS (all under-five deaths).

District	Ratio of early neonatal to neonatal deaths		Ratio of neonatal to under-five deaths	
	RRT Survey	DHS 2010	RRT Survey	DHS 2010
**Balaka**	0.7511	0.7407	0.3144	0.2796
**Salima**	0.7171	0.6365	0.2759	0.3007
**Total Balaka and Salima**	0.7359	0.6784	0.2959	0.2918
**Total Malawi**	na	0.7207	na	0.2603

B.3. Mortality results

Figure B9 presents the trends in under-five mortality rate for Balaka, Salima, and for both districts combined. It shows two main features. First, it suggests that levels and trends in under-five mortality rates were similar in both districts. Second, under-five mortality appears to have declined sharply in these two districts between 2000 and 2006, and stalled thereafter at around 100 deaths per 1000 live births.

Figure B9: Trends in under-five mortality rates for Balaka, Salima and total

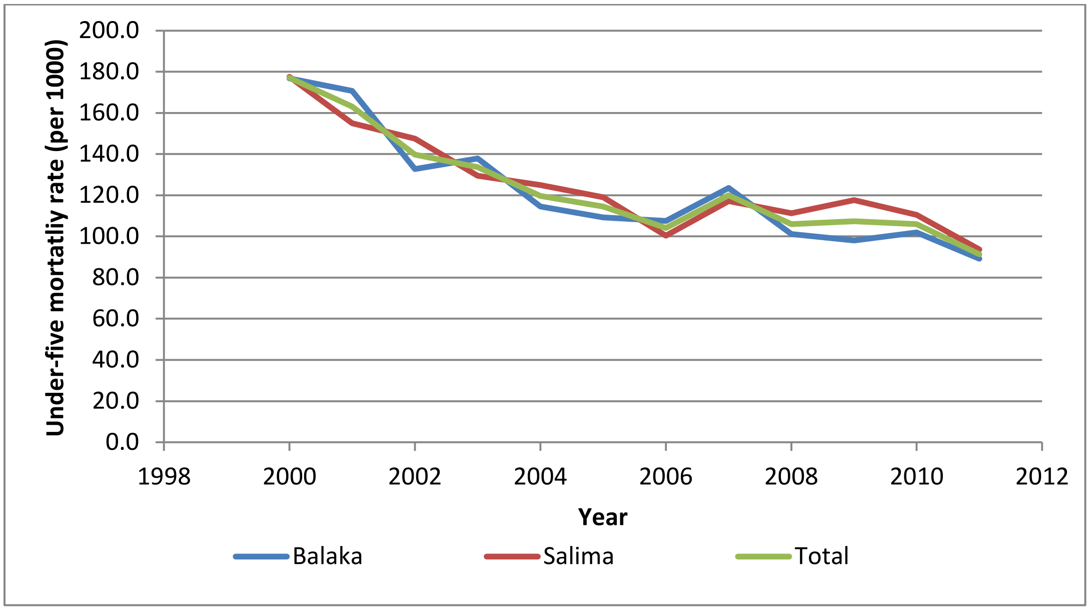

Figure B10: Trends in neonatal mortality rates for Balaka, Salima and total

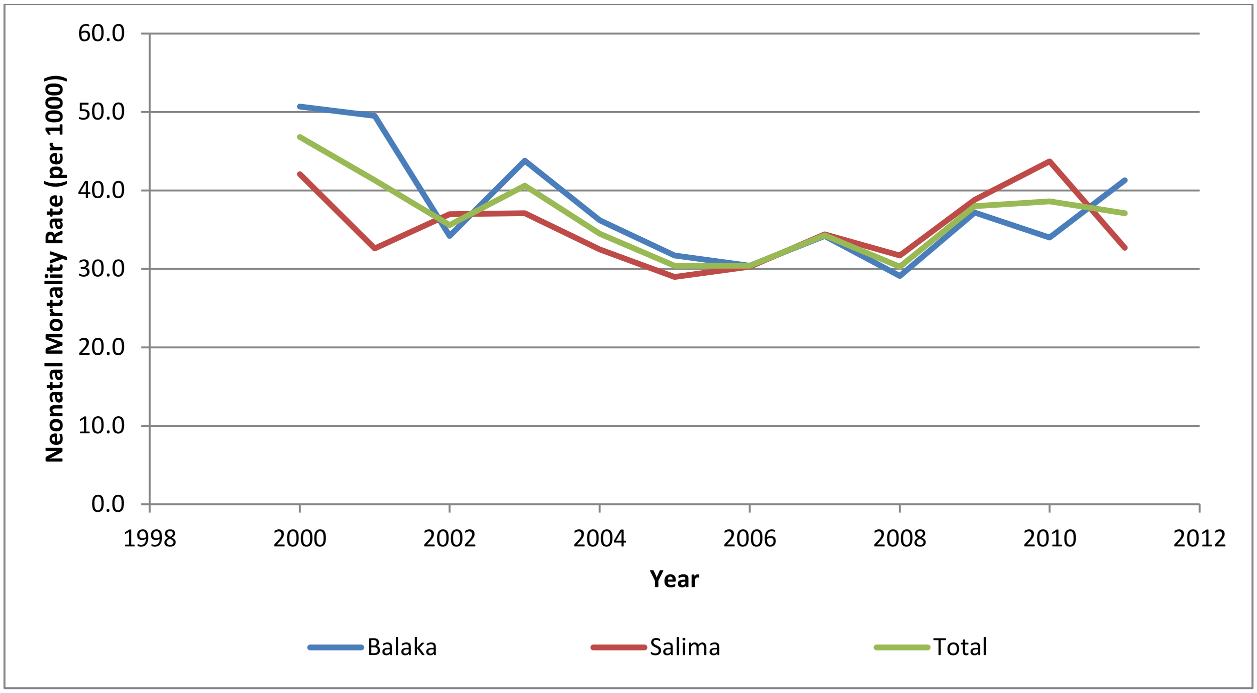

Table 10: Neonatal (NNMR), post-neonatal (PNNMR), infant (IMR), child (CMR) and under-five (U5MR) mortality rates by year some 2000 to 2011 for Balaka, Salima and total

Year	Balaka					Salima					Total				
	NN MR	PNN MR	IMR	CMR	U5MR	NN MR	PNN MR	IMR	CMR	U5MR	NN MR	PNN MR	IMR	CMR	U5MR
2011	41.3	23.6	64.9	25.9	89.1	32.7	28.6	61.3	34.5	93.7	37.1	26.0	63.1	30.1	91.3
2010	34.0	33.6	67.7	36.8	101.9	43.7	27.5	71.1	42.3	110.4	38.6	30.7	69.3	39.4	105.9
2009	37.2	25.0	62.2	38.2	98.0	38.8	31.0	69.8	51.4	117.6	38.0	28.0	65.9	44.3	107.3
2008	29.1	31.0	60.1	43.7	101.1	31.7	34.3	65.9	48.4	111.2	30.3	32.5	62.8	46.0	105.9
2007	34.2	24.2	58.3	69.3	123.6	34.4	35.0	69.4	51.2	117.1	34.3	29.3	63.7	60.8	120.0
2006	30.4	32.2	62.6	48.0	107.5	30.3	25.6	55.9	47.0	100.3	30.4	29.1	59.4	47.5	104.1
2005	31.7	31.9	63.6	48.8	109.3	29.0	35.7	64.7	59.0	119.0	30.4	33.8	64.3	53.7	114.5
2004	36.2	28.2	64.4	53.6	114.5	32.5	44.4	77.0	52.5	125.0	34.5	35.7	70.2	53.1	119.6
2003	43.8	39.6	83.4	59.4	137.9	37.1	32.8	70.0	64.0	129.5	40.6	36.4	77.0	61.3	133.6
2002	34.2	42.6	76.9	60.6	132.8	37.0	41.3	78.3	75.1	147.5	35.6	42.0	77.5	67.6	139.8
2001	49.5	52.1	101.6	76.9	170.7	32.6	45.7	78.3	83.7	155.0	41.3	49.2	90.5	79.7	163.0
2000	50.7	43.2	93.9	91.4	176.7	42.1	56.6	98.7	87.5	177.5	46.8	49.4	96.2	89.5	177.1

B.4. Conclusions about the gold-standard survey

The analysis of the gold-standard survey suggests that the survey is of good quality to support validation of the RRT methods. Analyses indicate levels and trends of under-five mortality rate that are similar in the two RRT districts. Under-five mortality rate was estimated at 102 in Balaka and 110 in Salima in 2010. The rate appeared to have decline rapidly between 2000 and 2006 and stalled since 2006 in both districts. Neonatal mortality was lower in Balaka (34 per 1000) compared to Salima (44 per 1000) in 2010. It also appeared to have decline between 2000 and 2006 but seemed to be increasing since 2006 in both district.

<u>APPENDIX 5</u>: Numbers of births and deaths reported in health facilities.

Table A5.1: Health facility births by month and year according to sex in Balaka and Salima

Year and month	District						Total		
	Balaka			Salima					
	Males	Females	Total	Males	Females	Total	Males	Females	Total
**2010**									
Jan	450	507	957	588	576	1164	1038	1083	2121
Feb	374	375	749	500	500	1000	874	875	1749
Mar	381	373	754	485	514	999	866	887	1753
Apr	394	337	731	501	510	1011	895	847	1742
May	408	410	818	537	511	1048	945	921	1866
Jun	378	402	780	504	513	1017	882	915	1797
Jul	433	452	885	580	530	1110	1013	982	1995
Aug	449	554	1003	612	603	1215	1061	1157	2218
Sep	531	552	1083	617	599	1216	1148	1151	2299
Oct	446	498	944	636	589	1225	1082	1087	2169
Nov	474	425	899	569	579	1148	1043	1004	2047
Dec	474	493	967	674	671	1345	1148	1164	2312
Total	5192	5378	10570	6803	6695	13498	11995	12073	24068
**2011**									
Jan	410	376	786	600	582	1182	1010	958	1968
Feb	349	332	681	478	470	948	827	802	1629
Mar	397	382	779	507	532	1039	904	914	1818
Apr	397	397	794	515	543	1058	912	940	1852
May	401	386	787	527	552	1079	928	938	1866
Jun	389	402	791	567	548	1115	956	950	1906
Jul	411	447	858	557	553	1110	968	1000	1968
Aug	445	518	963	618	597	1215	1063	1115	2178
Sep	447	461	908	597	619	1216	1044	1080	2124
Oct	374	360	734	508	531	1039	882	891	1773
Nov	375	382	757	530	515	1045	905	897	1802
Dec	441	393	834	561	580	1141	1002	973	1975
Total	4836	4836	9672	6565	6622	13187	11401	11458	22859

Table A5.2: Health facility under-five deaths by month and year according to sex in Balaka and Salima

Year and month	District						Total		
	Balaka			Salima					
	Males	Females	Total	Males	Females	Total	Males	Females	Total
2010									
Jan	12	11	23	33	27	60	45	38	83
Feb	12	8	20	22	26	48	34	34	68
Mar	13	6	19	23	17	40	36	23	59
Apr	14	18	32	24	23	47	38	41	79
May	7	11	18	19	7	26	26	18	44
Jun	8	5	13	20	15	35	28	20	48
Jul	18	13	31	22	24	46	40	37	77
Aug	12	10	22	22	18	40	34	28	62
Sep	17	17	34	28	17	45	45	34	79
Oct	12	13	25	17	18	35	29	31	60
Nov	14	13	27	28	27	55	42	40	82
Dec	33	18	51	17	16	33	50	34	84
Total	172	143	315	275	235	510	447	378	825
2011									
Jan	23	28	51	38	31	69	61	59	120
Feb	20	26	46	23	20	43	43	46	89
Mar	23	27	50	22	8	30	45	35	80
Apr	21	21	42	14	15	29	35	36	71
May	15	18	33	16	20	36	31	38	69
Jun	17	20	37	16	23	39	33	43	76
Jul	22	15	37	13	13	26	35	28	63
Aug	35	18	53	13	13	26	48	31	79
Sep	29	34	63	14	20	34	43	54	97
Oct	22	20	42	25	24	49	47	44	91
Nov	17	18	35	22	17	39	39	35	74
Dec	30	19	49	22	17	39	52	36	88
Total	274	264	538	238	221	459	512	485	997
